# Extension of an ICU-based noninvasive model to predict latent shock in the emergency department: an exploratory study

**DOI:** 10.3389/fcvm.2024.1508766

**Published:** 2024-12-23

**Authors:** Mingzheng Wu, Shaoping Li, Haibo Yu, Cheng Jiang, Shuai Dai, Shan Jiang, Yan Zhao

**Affiliations:** Emergency Center, Hubei Clinical Research Center for Emergency and Resuscitaion, Zhongnan Hospital of Wuhan University, Wuhan, Hubei, China

**Keywords:** shock, emergency department, intensive care unit, artificial intelligence, model

## Abstract

**Background:**

Artificial intelligence (AI) has been widely adopted for the prediction of latent shock occurrence in critically ill patients in intensive care units (ICUs). However, the usefulness of an ICU-based model to predict latent shock risk in an emergency department (ED) setting remains unclear. This study aimed to develop an AI model to predict latent shock risk in patients admitted to EDs.

**Methods:**

Multiple regression analysis was used to compare the difference between Medical Information Mart for Intensive Care (MIMIC)-IV-ICU and MIMIC-IV-ED datasets. An adult noninvasive model was constructed based on the MIMIC-IV-ICU v3.0 database and was externally validated in populations admitted to an ED. Its efficiency was compared with efficiency of testing with noninvasive systolic blood pressure (nSBP) and shock index.

**Results:**

A total of 50,636 patients from the MIMIC-IV-ICU database was used to develop the model, and a total of 2,142 patients from the Philips IntelliSpace Critical Care and Anesthesia (ICCA)-ED and 425,087 patients from the MIMIC-IV-ED were used for external validation. The modeling and validation data revealed similar non-invasive feature distributions. Multiple regression analysis of the MIMIC-IV-ICU and MIMIC-IV-ED datasets showed mostly similar characteristics. The area under the receiver operating characteristic curve (AUROC) of the noninvasive model 10 min before the intervention was 0.90 (95% CI: 0.84–0.96), and the diagnosis accordance rate (DAR) was above 80%. More than 80% of latent shock patients were identified more than 70 min earlier using the noninvasive model; thus, it performed better than evaluating shock index and nSBP.

**Conclusion:**

The adult noninvasive model can effectively predict latent shock occurrence in EDs, which is better than using shock index and nSBP.

## 1 Introduction

Latent shock is characterized by the presence of circulatory failure and is a common occurrence in critical illness. Approximately 30% of intensive care unit (ICU) patients suffer hemodynamic change, and the mortality rate is above 40% (1, 2). Most cases of latent shock can be reversed in the early stage of circulatory failure, especially prior to ICU transfer. However, timely identification of latent shock remains a great challenge.

Unlike the ICU, the emergency department (ED) manages a wide array of illnesses with unknown origins. Patients deemed to be in critical condition are promptly cared for by continuous monitoring of their circulatory function. The low nurse-to-patients ratios make manual assessments in Eds difficult, thus there is an excessive reliance on alarms for physiological measurements to identify individuals at risk of circulatory deterioration. These signals fail to incorporate comprehensive patient information, possibly causing non-specific alarms that contribute to alarm fatigue (3–5). Emergency physicians are engaged in the subsequent diagnostic and therapeutic processes, such as documenting medical records, conducting ultrasound examinations, or carrying out other invasive operations. Therefore, changes in monitoring data and laboratory results may not sent, interpreted, or acted upon by physicians in a timely manner (6, 7). A single measurement cannot fully describe the entire patient state and may lead to misunderstanding of the circulatory function. Integrated evidence analysis potentially decreases the incidence of misdiagnosis and adverse events, thereby improving patient safety and outcomes. In a high-paced environment like an ED, quickly filtering the important information from the vast amounts of data is necessary but increasingly hard for emergency health workers.

Machine-learning (ML) models utilize algorithms to learn from larger datasets and make predictions or decisions based on new data. Multiple parameter systems were developed as a method to identify patients at risk of delayed septic shock in EDs (8). The newly proposed hemodynamic stability index (HSI) model has outperformed against every single parameter for risk prediction in both adults and pediatrics (9, 10). The model consisted of more than thirty input features, including vital signs, laboratory measurements, and ventilation settings. Most of the variables are not routinely measured in EDs, and variables collected before ICU admission and in the first 6 h after ICU transfer were also excluded in these studies. More than 7,000 ICU transfers from the ED in Zhongnan Hospital of Wuhan University were retrospectively reviewed. It was found that the median interval from ED admission to ICU transfer was 5 h, with cases of latent shock mostly receiving fluid resuscitation within 6 h. How to quickly predict latent shock in cases within the ED remains a challenge.

The ICU-based noninvasive model for predicting latent shock risk has not yet been generalized to the ED. Non-invasive features that are easy to acquire in a short time should be considered. The study aimed to develop an adult noninvasive model in order to provide an earlier warning of latent shock risk, which is good for pre-hospital triage to the ICU.

## 2 Materials and methods

### 2.1 Definition of latent shock

Latent shock was defined as patients who were administrated with vasoactives and had a mean arterial pressure of below 65 mmHg (11). Fluid resuscitation was not included because most of the patients had a shorter ED stay once latent shock was identified. ED physicians are also more likely to use vasoactives than fluid resuscitation to improve the mean arterial pressure (MAP) before the underlying reasons for the condition are ascertained. Blood transfusion is time-consuming and rarely applied in the ED. More evidence of latent shock definition is described in the Supplementary Materials. Detailed categories or quantification of these definitions are listed in Supplementary Table S1.

### 2.2 Dataset selection

Medical Information Mart for Intensive Care (MIMIC) and eICU are two public datasets that are frequently used for ML research. Variables in the eICU dataset such as medicines or fluid administration are not labeled with the specific time. This makes it inconvenient for researchers to calculate the total volume of fluid infusion throughout a specific duration. Therefore, the Mimic-IV-ICU v3.0 dataset was used for model establishment between 2008 and 2022. In addition, data for external validation were extracted from two databases: the Philips IntelliSpace Critical Care and Anesthesia (ICCA) systems from the ED of Zhongnan Hospital of Wuhan University from December 2022 to July 2023 and the MIMIC-IV-ED between 2008 and 2022. Patients of an age ≥18 years were retrospectively included. Based on the unique patient number, in cases where the same patient is admitted repeatedly, only the first admission number was selected. Patients younger than 18 years of age, those with missing age values, those with stays of less than 30 min, or those with latent shock occurring within 30 min were excluded. All records in this study were strictly privacy-protected, and the use of the database was approved by the Beth Israel Deaconess Medical Center (BIDMC) Institutional Review Committee, Massachusetts Institute of Technology (CITI certificate number: 55436196) and Ethics Committee of Zhongnan Hospital of Wuhan University (2024066K).

### 2.3 Data processing and feature selection

Patients who received clinical intervention were placed in the unstable group. The start time of treatment was used as the time of diagnosis. The most recent feature values prior to the diagnosis of latent shock were extracted. For patients in the ED, missing values were filled in with the most recent data values. If clinical interventions were not received, patients were placed in the stable group, and any value that could be the result of the first measurement was extracted.

Features were screened based on missing values being less than 20%. The selected features were present in both databases, and the unit conversion was based on the ICCA system data. All variables were subjected to a rationality filter (Supplementary Table S2) to check whether their values were within the physiological validity range and to exclude outliers. By using random forests, the importance of model features in predicting latent shock was calculated. Features were input into the XGBoost classifier to get the SHAP value and force plot.

### 2.4 Algorithm selection

For the training set, 70% of the sample was randomly selected; the remaining 30% was used as the test set. The parameters were iteratively adjusted to achieve the best performance of the model. Several commonly used algorithms include random forest, logistic regression, adaptive boosting (AdaBoost), extreme gradient boosting (XGBoost), and neural networks. The parameters of these algorithms were iteratively tuned on the training set using five-fold internal cross-validation. The AUROC performance of these five algorithms was compared on the training set and the test set respectively.

### 2.5 Model development and external validation

A noninvasive model was constructed using features of greater than 0.01 importance that met the criteria. In EDs, identifying individuals at a high risk of latent shock without performing time-consuming laboratory tests is critical. Hence, noninvasive features were also included to build a noninvasive prediction model. After training, validating, and testing through common algorithms, the dataset was further divided through random sampling without replacement at a ratio of 7:3. The algorithm that worked best was selected to build the model and complete the external validation. The predictive accuracy of the model was interpreted based on the results of the calibration curve. If the calibration curve was close to the diagonal line, it indicated that the predicted probability of the model was consistent with the actual probability, and the model had a good calibration degree.

### 2.6 Statistical analysis

Continuous variables were presented as mean (standard deviation, SD) or median (interquartile range, IQR). Categorical variables were summarized by number (proportion). The unpaired *t*-test or the Mann–Whitney *U* test was used for continuous variables, and the Chi-square test or the Fisher exact test was used for categorical variables, as appropriate. In Python, functions were implemented to compute 95% confidence intervals (CI) for various metrics, including diagnosis accordance rate(DAR), area under the receiver operating characteristic curve (AUROC), sensitivity, specificity, F1 score, positive predictive value (PPV), and negative predictive value (NPV). For the shock index and the systolic blood pressure, the AUC was calculated using a binary logistic regression model. One-way analysis of variance was used to compare the AUC values of three models. Multiple regression analysis was used to compare the difference between the MIMIC-IV-ICU and MIMIC-IV-ED data sets. All statistical analyses were performed using the EmpowerStats statistical package (http://www.empowerstats.com, X&Y Solutions, Inc., Boston, MA) and R version 3.6.0. A two-sided *P* < 0.05 was considered statistically significant.

## 3 Results

### 3.1 Study population

**Figure 1:**
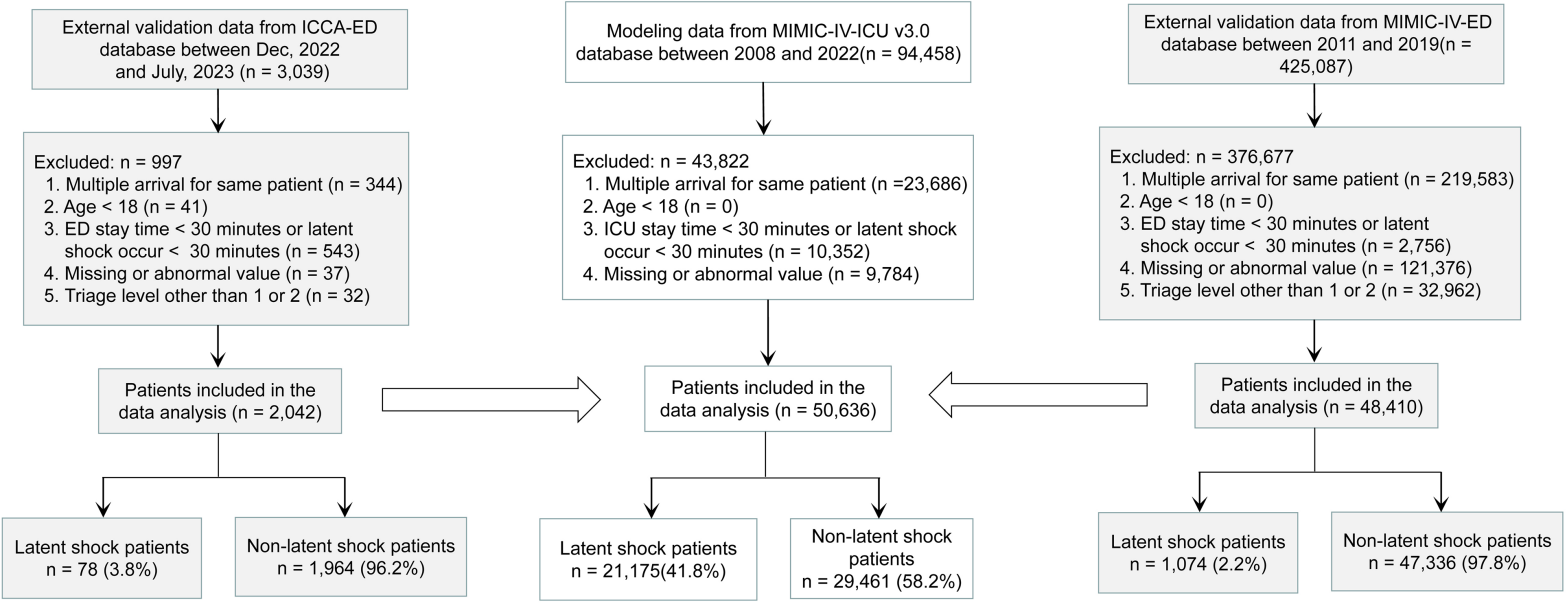
Study Flowchart. The stability and generalization ability of the model were verified externally several times. First, the MIMIC-IV-ED dataset is used for the first external validation, and then the ICCA-ED dataset is used for dynamic external validation every 10 minutes. MIMIC-IV-ICU, Medical Information Mart for Intensive Care IV in Intensive Care Unit; MIMIC-IV-ED, Medical Information Mart for Intensive Care IV in Emergency Department; ICCA-ED, IntelliSpace Critical Care and Anesthesia in Emergency Department; ICU, Intensive Care Unit; ED, Emergency Department.

A total of 94,458 patients were extracted from MIMIC-IV-ICU. A total of 43,822 patients were excluded, for reasons including repeated admission (23,686), ICU stay time <30 min or latent shock occurring <30 min (10,352), missing values (9,729), or abnormal values (55). Finally, 50,636 patients with latent shock (21,175) and non-latent shock (29,461) were included for model establishment. 425,087 patients were also extracted from the MIMIC-IV-ED. Ultimately, a total of 48,410 patients including latent shock (1,074) and non-latent shock group (47,336) were included for external validation on zero minute. 3,039 patients were also extracted from the ICCA system. Ultimately, a total of 2,142 patients including latent shock (78) and non-latent shock group (1,964) were included for external validation every 10 min (Figure 1). The modeling and validation data showed that the non-invasive feature distribution of the unstable group and the stable group were roughly similar (Table 1). The results of the multiple regression analysis between the MIMIC-IV-ICU and MIMIC-IV-ED datasets showed that most of the characteristics were similar (Supplementary Table S3). With an alert every 10 min, the 2,042 patients' vital signs were constantly changing. Patient information and characteristics of externally validated data on minute 0 are presented in Supplementary Table S4.

**Table 1:** Feature comparison between MIMIC-IV-ICU and MIMIC-IV-ED.

	Latent shock	Non-latent shock	*P*-values
MIMIC-IV-ICU			
Patients, *N* (%)	29,461 (41.8%)	21,175 (58.2%)	
Age, year, Median (Q1, Q3)	69.9 (57.3, 81.2)	63.3 (50.9, 75.0)	<0.001
Gender (Male), *N* (%)	10,589 (50.0%)	17,165 (58.3%)	<0.001
heart rate (HR, beats per minute), Median (Q1, Q3)	82.0 (70.0, 96.0)	85.0 (73.0, 100.0)	<0.001
Respiration rate (RR, beats per minute), Median (Q1, Q3)	19.0 (15.0, 22.0)	18.0 (15.0, 22.0)	<0.001
Transcutaneous Oxygen Saturation (SpO_2_,%), Median (Q1, Q3)	97.0 (95.0, 99.0)	98.0 (95.0, 100.0)	<0.001
Non, invasive systolic blood pressure (nSBP, mmHg), Median (Q1, Q3)	98.0 89.0, 109.0)	130.0 (116.0, 46.0)	<0.001
<90, *N* (%)	5,431 (25.6%)	485 (1.6%)	<0.001
≥90, *N* (%)	15,744 (74.4%)	28,976 (98.4%)	
Non, invasive diastolic blood pressure (nDBP, mmHg), Median (Q1, Q3)	49.0 (44.0, 54.0)	75.0 (65.0, 85.0)	<0.001
<60, *N* (%)	18,369 (86.7%)	3,878 (13.2%)	<0.001
≥60, *N* (%)	2,806 (13.3%)	25,583 (86.8%)	
Non, invasive mean blood pressure (nMBP, mmHg), Median (Q1, Q3)	61.0 (57.0, 64.0)	89.0 (79.0, 100.0)	<0.001
< 65, *N* (%)	17,788 (84.0%)	790 (2.7%)	<0.001
≥65, *N* (%)	3,387 (16.0%)	28,671 (97.3%)	
Shock index, Median (Q1, Q3)	0.8 (0.7, 1.0)	0.7 (0.5, 0.8)	<0.001
MIMIC-IV-ED			
Patients, *N* (%)	1,074 (2.2%)	47,336 (97.8%)	
Age, year, Median (Q1, Q3)	69.0 (55.0, 80.0)	63.0 (47.0, 76.0)	<0.001
Gender (Male), *N* (%)	479 (44.6%)	24,660 (52.1%)	<0.001
Heart rate (HR, beats per minute), Median (Q1, Q3)	83.0 (68.0, 98.0)	82.0 (70.0, 96.0)	0.475
Respiration rate (RR, beats per minute), Median (Q1, Q3)	18.0 (16.0, 22.0)	18.0 (16.0, 19.0)	<0.001
Transcutaneous Oxygen Saturation (SpO_2_,%), Median (Q1, Q3)	98.0 (96.0, 100.0)	98.0 (97.0, 100.0)	<0.001
Non, invasive systolic blood pressure (nSBP, mmHg), Median (Q1, Q3)	91.0 (84.0, 98.0)	132.0 (117.0, 148.0)	<0.001
<90, *N* (%)	457 (42.6%)	463 (1.0%)	<0.001
≥90, *N* (%)	617 (57.4%)	46,873 (99.0%)	
Non, invasive diastolic blood pressure (nDBP, mmHg), Median (Q1, Q3)	45.0 (41.0, 49.0)	75.0 (66.0, 85.0)	<0.001
<60, *N* (%)	1,039 (96.7%)	6,350 (13.4%)	<0.001
≥60, *N* (%)	35 (3.3%)	40,986 (86.6%)	
Non, invasive mean blood pressure (nMBP, mmHg), Median (Q1, Q3)	61.0 (57.0, 63.0)	94.0 (84.0, 105.0)	<0.001
<65, *N* (%)	955 (88.9%)	637 (1.3%)	<0.001
≥65, *N* (%)	119 (11.1%)	46,699 (98.7%)	
Shock index, Median (Q1, Q3)	0.9 (0.7, 1.1)	0.6 (0.5, 0.8)	<0.001

MIMIC-IV-ICU v3.0 database is from to 2008 to 2022. MIMIC-IV-ED database is from 2008 to 2019. Q1: the first quartile; Q3: the third quartile; *P*-values was calculated using non, parametric tests or Chi, square tests based on variable type.

### 3.2 Feature selection

**Figure 2:**
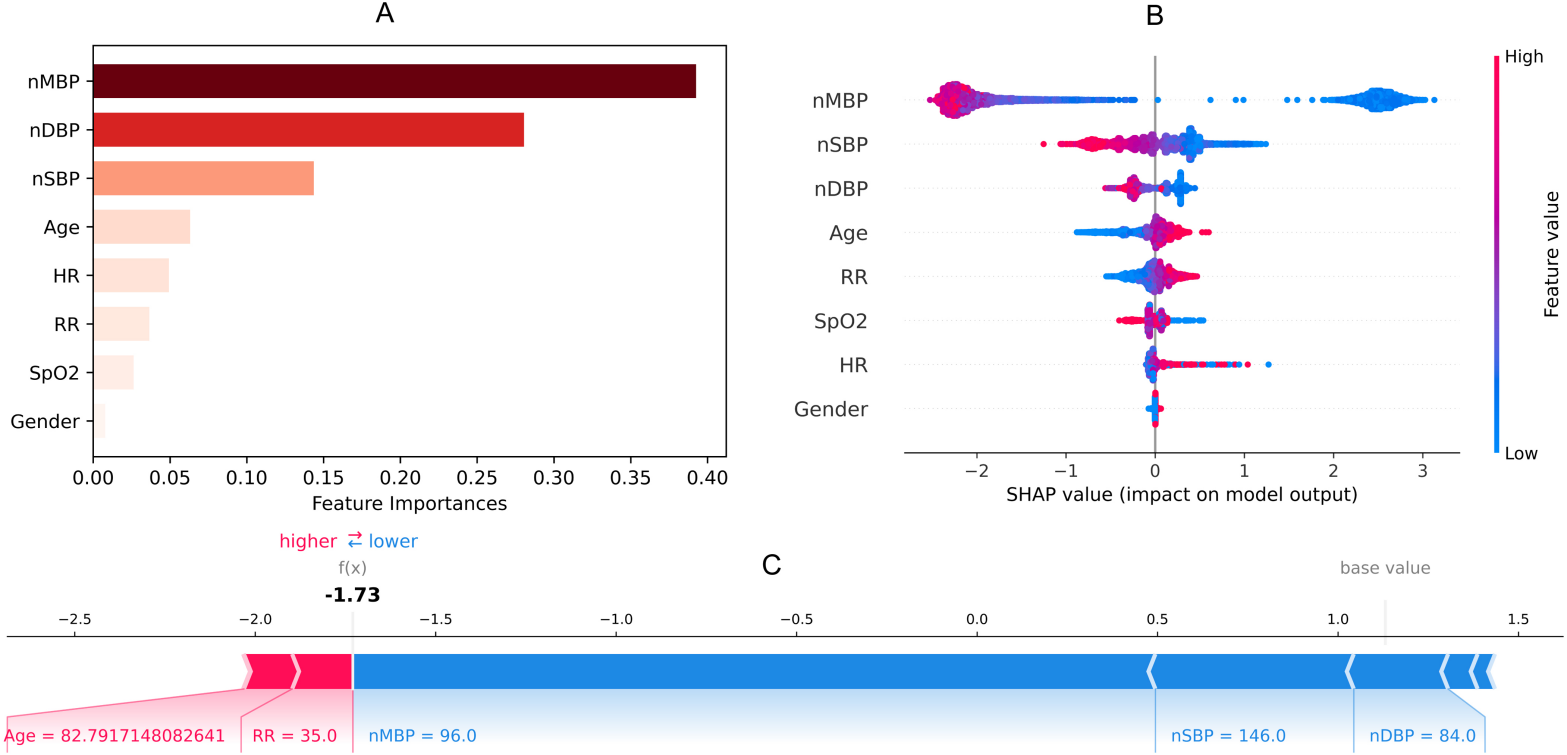
Feature importance **(A)**, SHAP value **(B)** and force plot **(C)** of noninvasive model for predicting latent shock. We found that the gender of the patient has very little effect and blood pressure has the greatest influence on noninvasive model. nSBP, noninvasive systolic blood pressure; nDBP, noninvasive diastolic blood pressure; nMBP, noninvasive mean blood pressure; HR, heart rate; RR, respiratory rate; SpO2, saturation of peripheral oxygen; SHAP, SHapley additive exPlanations.

Blood gas analysis features missing more than 60% were not collected. Finally, eight noninvasive features with relatively complete information were collected (Table 1). Temperature was not included in the external validation data due to this not being present in ED data. By using a random forest, Figure 2 shows the importance of the features (>0.01) of the model for predicting latent shock, finding that the gender of the patient has very little effect and blood pressure has the greatest influence on predicting latent shock. The higher the ranking, the more important the feature. The dot to the left of the digital baseline represents a negative contribution to experiencing latent shock, while the dot to the right represents a positive contribution. The farther away from the baseline, the greater the effect. Red stripes represent positive contributions and blue stripes represent negative contributions. The wider the stripes, the greater the contribution.

### 3.3 XGBoost algorithm

**Figure 3:**
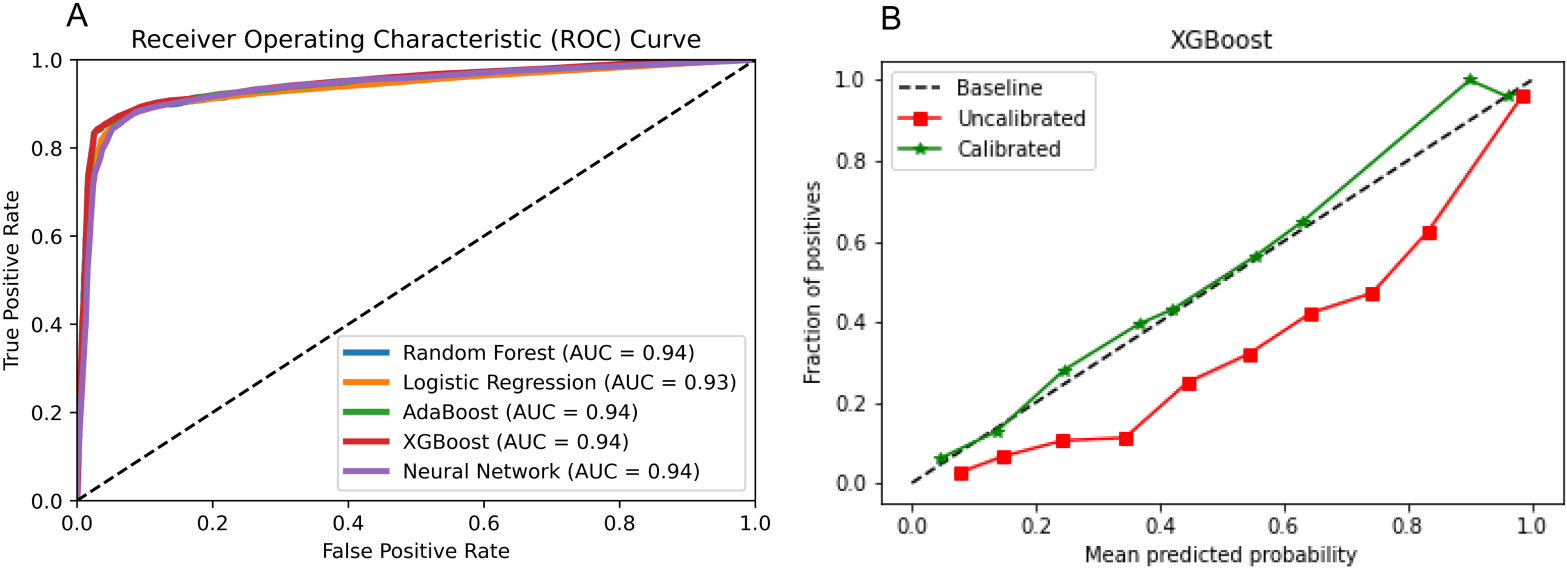
Algorithm selection. **(A)** Five algorithms for constructing the prediction model of latent shock; **(B)** The noninvasive model has a good calibration degree with XGBoost algorithm.

On the test set, Figure 3 shows that XGBoost is the best algorithm for constructing the prediction model of latent shock (AUC = 0.94). On the external validation, XGBoost algorithm was used to validate the performance of the noninvasive model. Figure 3 shows that the noninvasive model has a good calibration degree with XGBoost algorithm, which allows missing values in external validation.

### 3.4 Model performance over time

**Figure 4:**
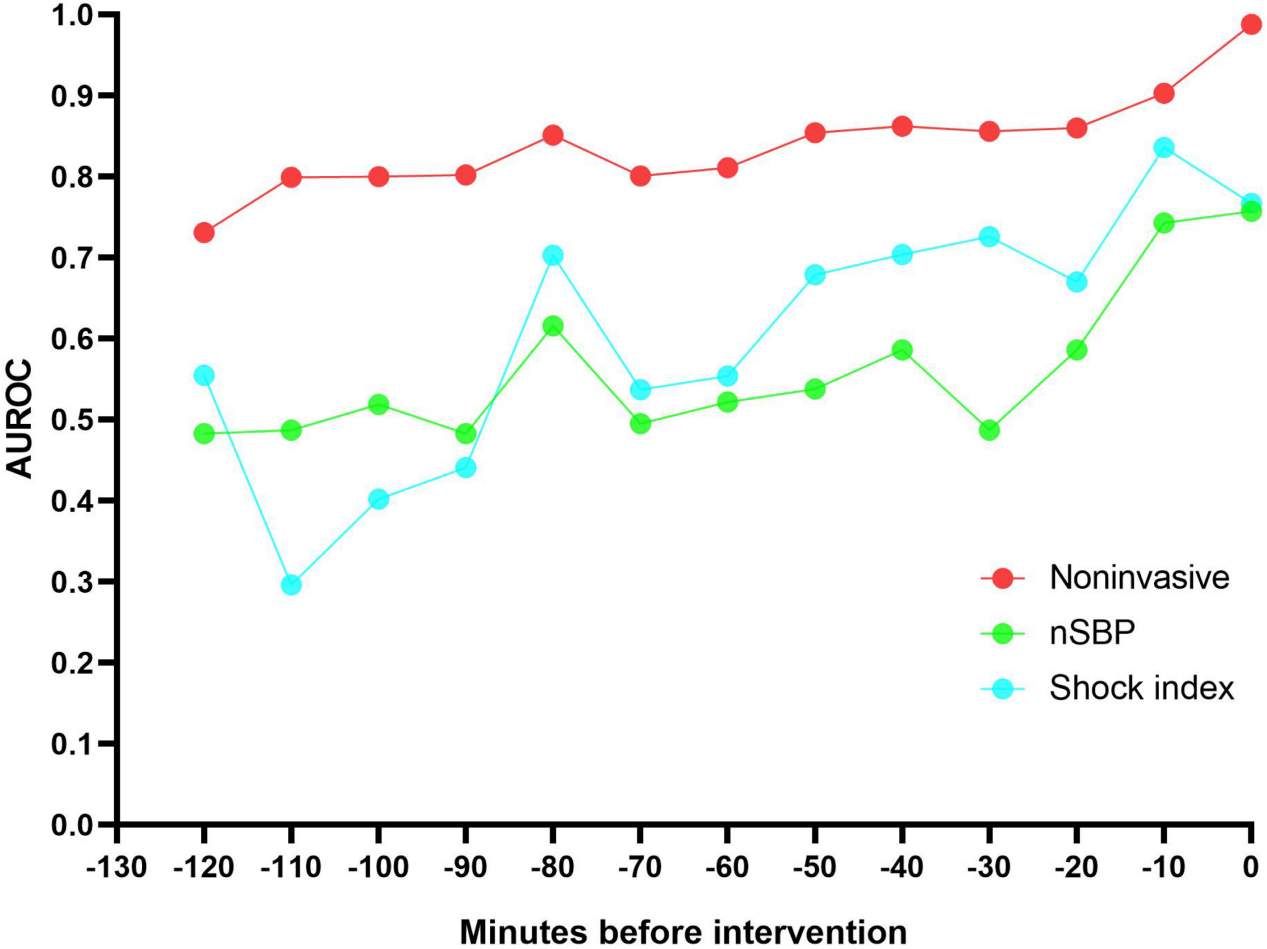
The non-invasive models had a higher AUROC 120 minutes before intervention than the shock index and noninvasive systolic blood pressure (nSBP). More than 80% of latent shock patients could be identified more than 70 minutes earlier.

Different thresholds cause model effects to vary. The results of model performance over time when the threshold is 0.2 or 0.4 are shown in Table 2. External validation results of the two datasets show that AUROC of the non-invasive model is as high as 0.99 at 0 min. AUROC of the noninvasive model 10 min before the intervention was 0.90 (95% CI: 0.84–0.96), and the DAR was more than 80%. The calibration plot also indicated that when the threshold was set to 0.2, more than 80% of latent shock patients could be identified more than 70 min earlier (Figure 4). A logistic regression model was used to calculate the area under the AUROC curve for shock index and noninvasive systolic blood pressure (nSBP). The non-invasive models had higher AUROC than the shock index and nSBP models. There were statistically significant differences in the AUC per 10 min of external validation among the three models (*P* < 0.05).

**Table 2:** Performance of noninvasive model for predicting latent shock, mean (95% CI).

Minutes before the intervention	Threshold	DAR	AUROC	Recall	Specificity	NPV	PPV	F1-score
MIMIC-IV-ED^a^								
0	0.4	0.97 (0.97–0.98)	0.99 (0.98–0.99)	0.96 (0.95–0.97)	0.97 (0.97–0.98)	1.00 (1.00–1.00)	0.46 (0.44–0.47)	0.62 (0.60–0.64)
ICCA-ED^b^								
0	0.2	0.85 (0.79–0.92)	0.99 (0.97–1.00)	0.98 (0.96–1.00)	0.85 (0.79–0.91)	1.00 (0.99–1.00)	0.16 (0.12–0.20)	0.27 (0.22–0.33)
10	0.2	0.86 (0.78–0.93)	0.90 (0.84–0.96)	0.86 (0.79–0.93)	0.86 (0.78–0.93)	1.00 (0.98–1.00)	0.11 (0.08–0.15)	0.20 (0.15–0.25)
20	0.2	0.84 (0.78–0.91)	0.86 (0.80–0.92)	0.74 (0.66–0.82)	0.85 (0.78–0.91)	0.99 (0.97–1.00)	0.12 (0.09–0.15)	0.20 (0.16–0.25)
30	0.2	0.85 (0.78–0.91)	0.86 (0.79–0.92)	0.69 (0.61–0.76)	0.85 (0.79–0.92)	0.99 (0.97–1.00)	0.12 (0.09–0.15)	0.20 (0.16–0.25)
40	0.2	0.84 (0.77–0.91)	0.86 (0.79–0.93)	0.73 (0.64–0.81)	0.84 (0.77–0.91)	0.99 (0.97–1.00)	0.11 (0.08–0.14)	0.19 (0.14–0.23)
50	0.2	0.84 (0.76–0.92)	0.85 (0.78–0.93)	0.75 (0.66–0.84)	0.84 (0.77–0.92)	0.99 (0.97–1.00)	0.11 (0.08–0.14)	0.19 (0.14–0.24)
60	0.2	0.83 (0.75–0.92)	0.81 (0.72–0.90)	0.63 (0.56–0.76)	0.84 (0.75–0.92)	0.99 (0.97–1.00)	0.09 (0.06–0.12)	0.16 (0.11–0.21)
70	0.2	0.84 (0.74–0.93)	0.80 (0.70–0.90)	0.56 (0.44–0.67)	0.84 (0.75–0.94)	0.99 (0.96–1.00)	0.07 (0.04–0.10)	0.12 (0.08–0.17)
80	0.2	0.83 (0.73–0.93)	0.85 (0.76–0.94)	0.77 (0.66–0.88)	0.83 (0.73–0.93)	0.99 (0.97–1.00)	0.09 (0.06–0.13)	0.17 (0.11–0.22)
90	0.2	0.83 (0.72–0.94)	0.80 (0.68–0.92)	0.70 (0.57–0.83)	0.84 (0.73–0.95)	0.99 (0.97–1.00)	0.08 (0.04–0.11)	0.14 (0.09–0.20)
100	0.2	0.83 (0.71–0.95)	0.80 (0.67–0.93)	0.65 (0.50–0.79)	0.83 (0.71–0.95)	0.99 (0.96–1.00)	0.07 (0.04–0.10)	0.12 (0.07–0.18)
110	0.2	0.82 (0.67–0.97)	0.80 (0.65–0.95)	0.58 (0.41–0.75)	0.82 (0.68–0.97)	0.99 (0.96–1.00)	0.05 (0.02–0.08)	0.09 (0.04–0.14)
120	0.2	0.82 (0.62–1.00)	0.73 (0.52–0.95)	0.57(0.35–0.79)	0.82(0.63–1.00)	0.99(0.96–1.00)	0.03(0.01–0.05)	0.06(0.02–0.10)

^a^ The noninvasive model of potential shock was externally validated on 0 min with the data set derived from MIMIC-IV-ED.

^b^ The noninvasive model of potential shock was externally validated every 10 min with the data set derived from ICCA in ED.

## 4 Discussion

Notably, the modeling and validation data revealed similar non-invasive feature distributions. Multiple regression analysis of MIMIC-IV-ICU and MIMIC-IV-ED datasets showed mostly similar characteristics. Blood pressure was identified as the most influential feature in predicting latent shock. Furthermore, our noninvasive model demonstrated AUROC and DAR of above 0.80 for predicting latent shock 70 min before intervention, outperforming both the single shock index and nSBP models, with statistically significant differences observed in the AUC per 10 min of external validation. This study has important clinical significance for pre-hospital care and for ED to triage of ICU.

In the ED, not all patients are referred to the ICU. Doctors classify the severity of patients' conditions, especially those with latent shock. The triage and acuity scale is called the Emergency Severity Index (ESI) Five Level triage system (12). Level 1 and level 2 patients are likely to be admitted to the ICU (13). This study found that the noninvasive model was a model that could be useful. The AUROC of our noninvasive model is similar to models from Chiang Dung-Hung et al. (9) (AUROC = 0.81) and Potes Cristhia et al. (10) (AUROC = 0.76). According to Table 1, Supplementary Tables S3, S4, the differences in most features between the MIMIC-IV-ICU and ED datasets are not significant. Thus, it can be seen that it is theoretically feasible for us to use the data of latent shock patients in the ICU to establish a noninvasive predictive model and adjust the model parameters based on the severity of the disease to provide an earlier warning of latent shock patients in the ED.

Clinically, vital signs are important disease information. Our study classifies vital signs as noninvasive and found that blood pressure is the most influential feature in predicting latent shock. Noninvasive features are also covered, such as age, gender, saturation of peripheral oxygen (SpO_2_), and GCS. Systolic blood pressure features are most important in models predicting latent shock, which is consistent with the reported importance of features (9, 10). Chang, H et al. (11) used six noninvasive indicators (nSBP, nDBP, RR, pulse rate, temperature, and SpO_2_) to establish an emergency department latent shock warning model. At 3 h before latent shock, the predictive AUROC values of RNN, MLP, RF, and LR methods were 0.822, 0.841, 0.852, and 0.830, respectively. Our study shows that more than 80% of latent shock patients could be identified more than 70 min earlier. And the noninvasive model is better than the shock index or nSBP. Therefore, an ICU-based noninvasive model for identifying latent shock risk in the ED is theoretically feasible.

Laboratory measurements and respiratory setting indicators are mostly invasive. Combining the model with laboratory measurements and respiratory setting indicators is conducive to improving its sensitivity, specificity, and accuracy (2, 14). But as the waiting time is long and cost high for invasive features. sequential organ failure assessment(SOFA) score was also confirmed as a predictor of mortality in ICU patients (15). The SOFA score exhibited the highest accuracy in predicting hospital mortality of septic latent shock at 0.880, followed closely by the SOS score (0.878), modified early warning score (MEWS) (0.858), quick sequential organ failure assessment (qSOFA) score (0.847), and NEWS score (0.833) (16). But the SOFA score contains invasive features. So, our ICU-based noninvasive model is a model that can be chosen but needs further study.

This study demonstrates that the non-invasive model can provide an early warning of latent shock risk in the emergency department, 70 min ahead of the current time, which holds significant value as a reference for early diagnosis and treatment. When Philips' ICCA system issues an alert for latent shock risk during the rescue and observation process, medical staff can immediately prioritize the patient's condition and initiate corresponding diagnostic and treatment protocols. This facilitates rapid identification and management of latent shock symptoms, thereby reducing the incidence of misdiagnosis and missed diagnoses. Patients can receive treatment earlier, alleviating their pain and discomfort. Consequently, this approach enhances patient satisfaction and trust, fostering improved doctor-patient relationships. Future research should explore the integration of our model with other noninvasive indicators to further enhance prediction accuracy, while also considering the balance between invasiveness, cost, and practicality in clinical settings. Ultimately, our study contributes to the ongoing effort to optimize triage and management strategies for latent shock patients in the ED.

## 5 Limitations

This study has limitations. First, while common clinical indicators were used as features, other factors such as a patient's temperature and Glasgow score (GCS) may also have provided useful features. Second, other important features need to be added, and the noninvasive model needs to be continually optimized. Third, when interpreting blood pressure data within the model, it is essential to fully consider the patient's underlying conditions and reasons for admission. For instance, blood pressure levels may differ between elderly and younger patients, potentially impacting the model's predictive performance across different age groups. Fourth, given the limited number of cases in the current study, a substantial amount of external validation set data is planned to be collected in the future. This will enable us to conduct analyses on various patient subgroups, allowing for separate modeling and external validation tailored to each subgroup. Fifth, in the process of promoting the model, the differences in ICU and ED data from different sources may affect the stability and generalization ability of the model, which requires multi-center external validation. Sixth, the significant imbalance in sample size between the stable and unstable groups within the external validation set has led to prediction biases, risks of overfitting, distorted evaluation metrics, and decreased statistical significance. In our future prospective studies, the sample size of the unstable group within the external validation set will be increased to mitigate the issue of sample imbalance. Therefore, these predictive models require further optimization and prospective study. Seventh, there was no analysis of the potential impact on model performance evaluation, clinical alert accuracy, and patient treatment outcomes based on different underlying disease subgroups of patients. In the later stage, we will establish subgroup analysis for different underlying diseases, integrate it into the ICCA system, and intelligently match early warning models for different types of patients.

## 6 Conclusion

This study found that ICU-based noninvasive model can effectively predict latent shock risk in ED, which is better than using the simple shock index and nSBP. Further prospective multicenter studies are needed to generalize these models.

## Data Availability

The raw data supporting the conclusions of this article will be made available by the authors, without undue reservation.
