# Supplementary material for: Extension of an ICU-based noninvasive model to predict latent shock in the emergency department: an exploratory study
Source: Front Cardiovasc Med. 2024 Dec 23;11:1508766. doi: 10.3389/fcvm.2024.1508766 (PMC11701063; doi:10.3389/fcvm.2024.1508766)
Supplement: Supplementary file 1 [file Datasheet1.pdf]

TABLE S1. Criteria to annotate hemodynamic instability between Mimic-IV-ICU Cohort and ICCA Cohort

Mimic-IV-ICU Cohort*	ICCA Cohort**
**Inotropic and vasoactive drugs**	**Inotropic and vasoactive drugs**
1.Dobutamine	1. Dopamine
2.Dopamine	2. Epinephrine
3.Epinephrine	3. Levophed
4.Levophed	4. Isoproterenol
5.Neosynephrine	5. Norepinephrine
6.Norepinephrine	6. Vasopressin
8.Phenylephrine	
9.Vasopressin	
**Mean arterial pressure (MBP)**	**Mean arterial pressure (MBP)**
Two consecutive recordings of MBP < 65mmHg	Two consecutive recordings of MBP < 65mmHg

*Mimic-IV-ICU Cohort which was used to developed the model of adult hemodynamic instability (AHI)

**ICCA Cohort in ED for this AHI external validation study

TABLE S2. Clinical features

Feature name	Unit	Plausibility low	Plausibility high	Feature type
Age	years	18	140	noninvasive
Gender				noninvasive
non-invasive diastolic blood pressure(nDBP)	mmHg	0	250	noninvasive
non-invasive systolic blood pressure(nSBP)	mmHg	0	270	noninvasive
non-invasive mean blood pressure(nMBP)	mmHg	0	250	noninvasive
Heart rate(HR)	bpm	0	600	noninvasive
Respiration rate(RR)	times/min	0	50	noninvasive
Temperature	degrees (℃)	34	42	noninvasive
oxygen saturation(SpO2)	%	0	100	noninvasive

Table S3 Multivariate regression analysis of potential shock.

Characteristics	MIMIC-IV-ICU[a]		MIMIC-IV-ED[b]	
	HR (95% CI)	*P*-value	HR (95% CI)	*P*-value
Age (per year)	1.0 (1.0, 1.0)	< 0.001	1.0 (1.0, 1.0)	0.005
Gender (Male vs Female)	1.0 (0.9, 1.0)	0.316	1.0 (0.8, 1.2)	0.986
non-invasive diastolic blood pressure (nDBP, per mmHg)	1.0 (1.0, 1.0)	< 0.001	0.7 (0.6, 0.9)	0.004
non-invasive systolic blood pressure (nSBP, per mmHg)	1.0 (1.0, 1.0)	< 0.001	0.8 (0.7, 0.9)	<0.001
non-invasive mean blood pressure (nMBP, per mmHg)	0.9 (0.9, 0.9)	< 0.001	1.2 (0.9, 1.6)	0.241
heart rate (HR, beats per minute)	1.0 (1.0, 1.0)	< 0.001	1.1 (1.0, 1.1)	< 0.001
respiration rate (RR, beats per minute)	1.0 (1.0, 1.0)	< 0.001	1.1 (1.0, 1.1)	< 0.001
transcutaneous Oxygen Saturation (SpO2, %)	1.0 (1.0, 1.0)	< 0.001	1.1 (1.0, 1.1)	0.006
non-invasive shock index (nSI)	6.7 (3.3, 13.5)	< 0.001	0.0 (0.0, 0.0)	< 0.001

CI: confidence interval; *HR*: hazard ratio. The *P*-value was used to assess whether the strength of the association between risk factors and latent shock was statistically significant. Multivariate regression analysis are based on the complete cases without missing value. All factors were simultaneously put into the Multivariate regression model.

[a]Multivariate regression analyses are based on Medical Information Mart for Intensive Care (MIMIC) IV-ICU v3.0 dataset.

[b]Multivariate regression analyses are based on MIMIC-IV-ED dataset.

TABLE S4. Patient information and characteristics of externally validated data on 0 minute, Median (Q1, Q3)

	Latent Shock	Non-latent Shock	*P*-values
Patients, N	56	1956	
Age, year Median (Q1, Q3)	72.0 (62.5-83.0)	67.0 (56.0-76.0)	0.011
Gender (Male), N (%)	44 (78.6%)	1247 (63.8%)	0.023
heart rate (HR, beats per minute)	98.0 (83.0-117.5)	84.5 (72.0-97.8)	<0.001
respiration rate (RR, beats per minute)	23.0 (18.0-29.0)	19.0 (16.0-23.0)	<0.001

transcutaneous Oxygen Saturation (SpO2, %)	96.0 (91.0-99.0)	98.0 (96.0-100.0)	0.001
non-invasive systolic blood pressure (nSBP, mmHg)	87.0 (77.0-96.2)	130.0 115.0-149.0)	<0.001
non-invasive diastolic blood pressure (nDBP, mmHg)	50.5 (43.0-54.0)	78.0 (68.0-89.0)	<0.001
non-invasive mean blood pressure (nMBP, mmHg)	62.0 (57.0-64.0)	94.0 (83.0-106.0)	<0.001

Patient information and features from the Philips IntelliSpace Critical Care and Anesthesia (ICCA) systems in ED of Zhongnan Hospital of Wuhan University from December 2022 to July 2023; Q1: the first quartile; Q3: the third quartile; P-values was calculated using non-parametric tests or Chi-square tests based on variable type.
